# Associação do Valor Pan-Imune-Inflamatório com Desfechos de Longo Prazo na Insuficiência Cardíaca Agudamente Descompensada

**DOI:** 10.36660/abc.20230817

**Published:** 2024-07-01

**Authors:** Bektas Murat, Selda Murat, Mehmet Eren Altınbas, Halit Emre Yalvac, Fatih Enes Durmaz, Kadir Ugur Mert, Yüksel Cavusoglu

**Affiliations:** 1 Eskisehir City Hospital Department of Cardiology Eskisehir Turquia Eskisehir City Hospital, Department of Cardiology, Eskisehir – Turquia; 2 Eskisehir Osmangazi University Medicine Faculty Department of Cardiology Eskisehir Turquia Eskisehir Osmangazi University, Medicine Faculty Department of Cardiology, Eskisehir – Turquia; 3 Sivas Numune Hospital Department of Cardiology Sivas Turquia Sivas Numune Hospital, Department of Cardiology, Sivas – Turquia

**Keywords:** Mortalidade, Biomarcadores, Insuficiência Cardíaca Sistólica

## Abstract

**Fundamento:**

Embora tenha havido melhorias significativas no tratamento da insuficiência cardíaca (IC) nas últimas décadas, seu prognóstico permanece desfavorável. Embora existam muitos biomarcadores que podem ajudar a prever o prognóstico de pacientes com IC, há necessidade de biomarcadores mais simples, menos dispendiosos e mais facilmente disponíveis.

**Objetivo:**

Avaliar o valor preditivo do valor pan-imune-inflamatório (PIV, do inglês *pan-immune-inflammation value*) em pacientes com IC agudamente descompensada.

**Métodos:**

Analisamos 409 pacientes com IC com fração de ejeção reduzida internados por IC aguda descompensada. Os pacientes foram divididos em 3 grupos de acordo com os tercis de PIV: tercil 1 (PIV < 357,25), tercil 2 (PIV ≥ 357,25 e < 834,55) e tercil 3 (PIV ≥ 834,55). Foram considerados estatisticamente significativos valores de p < 0,05. Curvas de Kaplan-Meier e modelos de regressão de riscos proporcionais de Cox foram utilizados para avaliar a associação entre PIV e mortalidade por todas as causas. O desfecho primário foi mortalidade por todas as causas em 5 anos, e o desfecho secundário compreendeu a mortalidade por todas as causas intra-hospitalar em 30 dias, em 180 dias e em 1 ano

**Resultados:**

Mostramos que valores mais elevados de PIV estavam associados a desfechos primários e secundários. A curva de Kaplan-Meier mostrou que pacientes com valores mais elevados de PIV apresentaram risco aumentado de mortalidade por todas as causas em curto e longo prazo (log-rank p < 0,001). Na análise multivariada, o PIV foi identificado como um preditor independente de mortalidade por todas as causas em longo prazo em pacientes com IC aguda descompensada, e observamos um aumento de 1,96 vezes no risco de um evento (razão de chances: 1,96; intervalo de confiança de 95%: 1,330 a 2,908; p = 0,001).

**Conclusões:**

Nosso estudo mostrou que o novo biomarcador PIV pode ser usado como preditor de prognóstico em pacientes com IC aguda descompensada.

## Graphical abstract

**Figura Central:**
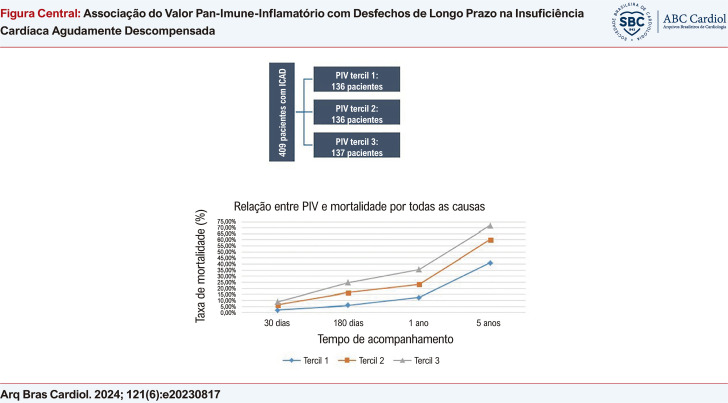
: Associação do Valor Pan-Imune-Inflamatório com Desfechos de Longo Prazo na Insuficiência Cardíaca Agudamente Descompensada

*Pacientes nos tercis de PIV e relação entre PIV e mortalidade por todas as causas. ICAD: insuficiência cardíaca agudamente descompensada; PIV: valor pan-imune-inflamatório.*

## Introdução

A insuficiência cardíaca (IC) é uma síndrome clínica acompanhada de sintomas como falta de ar, inchaço dos tornozelos, fadiga e tolerância à atividade diminuída, resultando em débito cardíaco reduzido decorrendo de comprometimento estrutural e/ou funcional do coração para manter a perfusão e as necessidades metabólicas de vários tecidos e órgãos.^1,2^ Embora melhorias nos tratamentos e suas implementações tenham melhorado a sobrevida e a hospitalização dos pacientes com insuficiência cardíaca com fração de ejeção reduzida (ICFEr) nos últimos 30 anos, continua a ser um grave problema de saúde pública e representa um sério fardo econômico, em relação ao aumento da expectativa de vida e ao envelhecimento da população global.^3,4^

A estimativa do prognóstico para desfechos clínicos, como morbidade, mortalidade e hospitalização, desempenha um papel importante para ajudar os pacientes, suas famílias e os médicos a decidirem sobre o tipo e o momento apropriados do tratamento (especialmente as decisões relativas à transição rápida para tratamentos adicionais). Nas últimas décadas, embora numerosos marcadores prognósticos tenham sido identificados para prever morte e/ou hospitalização por IC em diferentes populações de pacientes com IC, alguns deles são úteis na previsão do óbito, mas são insuficientes na previsão de hospitalizações. Além disso, a sua aplicabilidade clínica é limitada, e a estratificação precisa do risco na IC permanece difícil.^5-7^

Embora a patogênese específica da IC permaneça incerta, a ativação imunológica anormal e a inflamação crônica desempenham um papel importante, e tem sido provado que existe uma estreita relação entre a inflamação e doenças cardiovasculares.^8^ Estudos demonstraram que a inflamação desempenha um papel importante no início e na progressão da aterosclerose e está intimamente associada à patogênese da IC e à remodelação cardíaca.^9^ Evidências crescentes também propõem que as respostas imunológicas e inflamatórias podem desempenhar um papel patogênico no desenvolvimento da IC crônica.^10^

A IC aguda pode ser a primeira manifestação de IC (início novo) ou, mais frequentemente, pode ser devida à descompensação aguda da IC crônica. A IC agudamente descompensada é a principal causa de hospitalização em pessoas com mais de 65 anos e está associada a altas taxas de mortalidade e rehospitalização, com mortalidade intra-hospitalar de 4% a 10%. Em comparação com pacientes com IC aguda descompensada, pacientes com IC de início recente podem ter maior mortalidade intra-hospitalar, mas apresentam menores taxas de mortalidade pós-alta e de rehospitalização.^11^

Mais recentemente, um biomarcador de células inflamatórias extensas denominado valor pan-imune-inflamatório (PIV, do inglês *pan-immune-inflammation value*), que inclui contagens de neutrófilos, plaquetas, monócitos e linfócitos, demonstrou estar fortemente associado a desfechos piores e mortalidade em muitos tipos de câncer. Todos esses estudos mostraram que o PIV é um indicador de inflamação mais estável e melhor do que os biomarcadores imunológicos bem estabelecidos, como a relação neutrófilos/linfócitos (RNL) e a relação plaquetas/linfócitos (RPL).^12-14^

Portanto, o objetivo do presente estudo foi avaliar o valor prognóstico em longo prazo do PIV, um novo biomarcador de baixo custo que é facilmente disponível, contendo todos os componentes imunoinflamatórios obtidos do sangue periférico, em pacientes com IC agudamente descompensada.

## Métodos

### População do estudo

**Figura 1:**
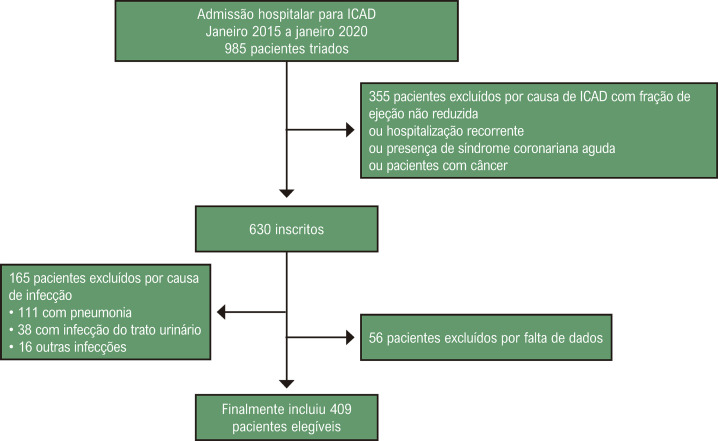
– Fluxograma para inclusão de pacientes. ICAD: insuficiência cardíaca agudamente descompensada.

O presente estudo retrospectivo incluiu um total de 409 pacientes hospitalizados por IC agudamente descompensada no nosso hospital terciário de referência entre janeiro de 2015 e janeiro de 2020. Incluímos pacientes maiores de 18 anos que foram admitidos no departamento de emergência e hospitalizados por ICFEr agudamente descompensada com base na definição das Diretrizes da Sociedade Europeia de Cardiologia.^3^ Os critérios de exclusão foram os seguintes: dispneia principalmente por causas não cardíacas, choque séptico, síndrome coronariana aguda, gestantes, pacientes com doenças inflamatórias ativas, pacientes com infecção ativa (pneumonia, infecção do trato urinário, etc.), pacientes com qualquer leucemia, pacientes sem parâmetros hematológicos e pacientes com IC hospitalizados principalmente por infecção (Figura 1).

### Coleta de dados

Foram coletados do sistema médico hospitalar os dados demográficos e o histórico médico dos pacientes, incluindo idade, sexo, hipertensão, diabetes mellitus, hiperlipidemia e tabagismo. No momento da admissão hospitalar, todas as amostras de sangue venoso rotineiramente mediram o seguinte: glicose, creatinina, colesterol, colesterol de lipoproteína de alta densidade, lipoproteína de baixa densidade, colesterol total, triglicerídeos, pico de creatinoquinase fração MB, pico de troponina T, a fração N-terminal do peptídeo natriurético do tipo B (NT-proBNP), hemoglobina, contagem de neutrófilos, contagem de linfócitos, contagem de monócitos e contagem de plaquetas. Todos os dados demográficos, médicos, ecocardiográficos e laboratoriais foram obtidos do banco de dados do hospital. As comorbidades foram aceitas como diagnósticos se encontradas no histórico médico do paciente ou nos registros e prontuários do paciente.

### Cálculo do valor pan-imune-inflamatório

O PIV foi calculado do modo seguinte: [contagem de neutrófilos (10^3^/μL) × contagem de plaquetas (10^3^/μL) × contagem de monócitos (10^3^/μL)]/contagem de linfócitos (10^3^/μL).^13^

### Desfechos do estudo

A mortalidade por todas as causas em longo prazo (5 anos) de pacientes com IC agudamente descompensada foi selecionada como desfecho primário, enquanto o desfecho secundário foi definido como mortalidade em curto prazo, incluindo a mortalidade por todas as causas intra-hospitalar, em 30 dias, em 180 dias e em 1 ano. O desfecho primário e secundário do acompanhamento foi obtido do banco de dados do hospital e por ligações telefônicas com os pacientes e/ou seus familiares.

O estudo recebeu aprovação do comitê de ética da nossa universidade.

### Análise estatística

A análise estatística foi realizada utilizando o IBM SPSS Statistics 21.0 (IBM Corp., lançado em 2012, IBM SPSS Statistics for Windows, versão 21.0. Armonk, NY, EUA).

Variáveis contínuas normalmente distribuídas são relatadas como média ± desvio padrão, enquanto variáveis distorcidas são expressas como medianas e intervalos interquartis. Foram realizados testes de Shapiro-Wilk e traçados mapas de densidade para determinar a normalidade da distribuição das variáveis contínuas. As variáveis categóricas foram expressas como frequências absolutas e relativas, e as associações relacionadas às variáveis categóricas foram verificadas por meio do teste qui-quadrado ou o teste exato de Fisher.

As comparações das diferenças entre os grupos foram realizadas por análise de variância (ANOVA unidirecional). Para análise post-hoc foi utilizado o teste de Tukey. Foi utilizado o teste H de Kruskal-Wallis para comparar os grupos que não se enquadraram na distribuição normal. Para análise post-hoc foi utilizado o teste de Dunn.

Os pacientes foram agrupados em 3 tercis com níveis aumentados de PIV para análise subsequente.

Foram aplicados modelos univariados e multivariados para prever a mortalidade por todas as causas. Variáveis com p < 0,05 no modelo univariado foram incluídas no modelo multivariado para avaliar os efeitos abrangentes do PIV no evento do desfecho. Também mostramos a relação entre o PIV e a sobrevida dos pacientes por meio da curva de Kaplan-Meier, utilizando o teste log-rank para testar hipóteses. Foram calculados o *odds ratio* (OR)e seu intervalo de confiança (IC) de 95%. Todas as comparações foram bicaudais, com p < 0,05 considerado significativo.

## Resultados

De acordo com os critérios de inclusão mencionados acima, 409 pacientes com diagnóstico de IC foram incluídos neste estudo. A média de idade foi de 62,2 ± 11,8 anos e houve predominância do sexo masculino, com 279 pacientes masculinos (68,2%). Os pacientes foram divididos em 3 grupos (tercil 1, tercil 2 e tercil 3) de acordo com os tercis do PIV (Figura Central). As características basais da população do estudo estão listadas na Tabela 1. Pacientes mais idosos apresentaram maior PIV (p = 0,038). Comparando as comorbidades entre os 3 grupos, o PIV não apresentou diferença estatística entre hipertensão e diabetes mellitus (p = 0,717 e p = 0,348, respectivamente). Pacientes com maior PIV foram associados a maiores taxas de insuficiência renal e doença pulmonar crônica (p = 0,002 e p = 0,004, respectivamente), e a prevalência de doença arterial coronariana foi menor no tercil mais alto de PIV (p = 0,021).

**Tabela 1:** – Características basais da população do estudo por grupos tercis de PIV

	PIV < 357,25 (n=136) (1)	PIV ≥ 357,25 e < 834,55 (n=136) (2)	PIV ≥ 834,55 (n=137) (3)	Valor p	Comparações múltiplas
Idade, anos	66,2±11,9	66,3±12,9	69,2±10,2	0,038	1-3: 0,041
Sexo feminino, n (%)	49 (36,0%)	37 (27,2 %)	44 (32,1%)	0,293	-
HT, n (%)	83 (61,0 %)	77 (56,60%)	83 (60,6%)	0,717	-
DM, n (%)	52 (38,2%)	62 (45,6%)	63 (46,0%)	0,348	-
DAC, n (%)	82 (60,3%)	103 (75,7%)	96 (70,1%)	0,021	-
DPC, n (%)	28(20,6%)	38(27,9%)	53 (38,7%)	0,004	-
FA, n (%)	30 (22,1%)	26 (19,4%)	48 (35,0%)	0,038	-
Insuficiência renal, n (%)	60 (44,1%)	67 (49,3%)	89,0 (65,0%)	0,002	-
FC, batimentos/minuto	72,9±14,7	77,2±19,1	83,5±18,5	<0,001	1-3: <0,001 2-3: 0,009
Sinais de hipoperfusão, n (%)	9(6,6%)	14(10,3%)	23(16,8%)	0,027	-
**Resultados laboratoriais**					
Hemoglobina, g/dl	12,9±2,07	12,3±2,0	11,9±1,8	<0,001	1-3: <0,001
Plaquetas, (10^3^ /mL)	181,0(15,25-217,75)	207,0(173,75-253,5)	250,0(194,0-303,5)	<0,001	1-2: 0,012 1-3: <0,001 2-3: <0,001
WBC, (10^3^/mL)	7,0(5,8-8,4)	8,1(6925-9,3)	10,2(8,8-12,25)	<0,001	1-2: 0,031 1-3: <0,001 2-3: <0,001
Linfócitos (10^3^ /mL)	1,7(1,4-2,2)	1,45(1,0-1,8)	1,2(0,95-1,6)	<0,001	1-2: <0,001 1-3: <0,001
Neutrófilos (10^3^ /mL)	4,25(3,5-5,3)	5,7(4,625-6,0)	7,90(6,55-9,70)	<0,001	1-2: 0,007 1-3: <0,001 2-3: <0,001
Monócitos (10^3^ /mL)	0,55(0,4250-0,70)	0,60(0,50-0,80)	0,90(0,70-1,10)	<0,001	1-3: <0,001 2-3: <0,001
LDL-C (mg/dL)	105(77,0-130,02)	95,0(73,0-121,0)	90,0(70,0-112,0)	0,010	1-3: 0,003
Triglicerídeos (mg/dL)	113,0(87,1-149,2)	108,0(77,65-155,25)	108(84,25-145,0)	0,778	NS
NT-proBNP, pg/ml	1828,0 (650,2-5732,0)	3949(1228,7-11308)	5108,0 (2615,2-14394,0)	<0,001	1-2: 0,016 1-3: <0,001
Albumina, g/dl	3,83±0,57	3,73±0,58	3,54±0,57	<0,001	1-3: <0,001 2-3: 0,028
PCR, (mg/dL)	5,82 (3,4-19,3)	13,65(5,64-32,5)	21,0(10,1-50,3)	<0,001	1-3: <0,001 2-3: 0,006
Creatinina, (mg/dL)	1,23±0,69	1,49±1,08	1,54±0,82	0,002	1-2: 0,044 1-3: 0,012
Glicose, (mg/dL)	107 (84-152)	112,0(88,0-153,0)	117(90,5-164,5)	0,809	NS
Sódio, (mmol/L)	139,1±3,9	138,7±4,2	136,4±5,6	<0,001	1-3: <0,001 2-3: <0,001
Potássio, (mmol/L)	4,54±0,5	4,50±0,49	4,48±0,59	0,676	NS
Troponina-T	0,022(0,013-0,05)	0,036(0,019-0,062)	0,045(0,024-0,098)	0,176	NS
FEVE, %	26,5±7,25	24,5±8,5	25,2±7,9	0,091	NS
PSAP, mm Hg	48,6±17,1	50,6±15,6	50,6±15,8	0,224	NS
**Desfechos (mortalidade por todas as causas)**					
Mortalidade em 30 dias	3 (2,2%)	9(6,6%)	12(8,8%)	<0,001	-
Mortalidade em 365 dias	17 (12,5%)	32(23,5%)	46(35,8%)	<0,001	-
Mortalidade em 5 anos	56 (41,2%)	82 (60,3%)	99 (72,3%)	<0,001	-

*DAC: doença arterial coronariana; DM: diabetes mellitus; DPC: doença pulmonar crônica; FA: fibrilação atrial; FC: frequência cardíaca; FEVE: fração de ejeção do ventrículo esquerdo; HT: hipertensão; LDL-C: colesterol de lipoproteína de baixa densidade; NS: não significativo; NT-proBNP: fração N-terminal do peptídeo natriurético do tipo B; PCR: proteína C reativa; PIV: valor pan-imune-inflamatório; PSAP: pressão sistólica da artéria pulmonar; WBC: contagem de leucócitos.*

Na população geral do estudo, o acompanhamento médio foi de 5 anos. Observamos um total de 56 eventos no tercil 1, 82 eventos no tercil 2 e 99 eventos no tercil 3 durante o acompanhamento.

**Figura 2:**
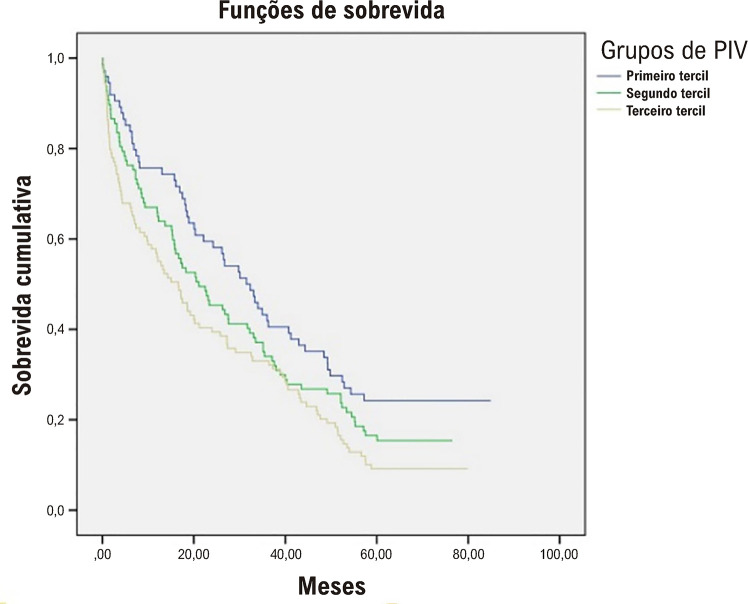
– Relação entre PIV e mortalidade por todas as causas por período de tempo. PIV: valor pan-imune-inflamatório.

**Figura 3:**
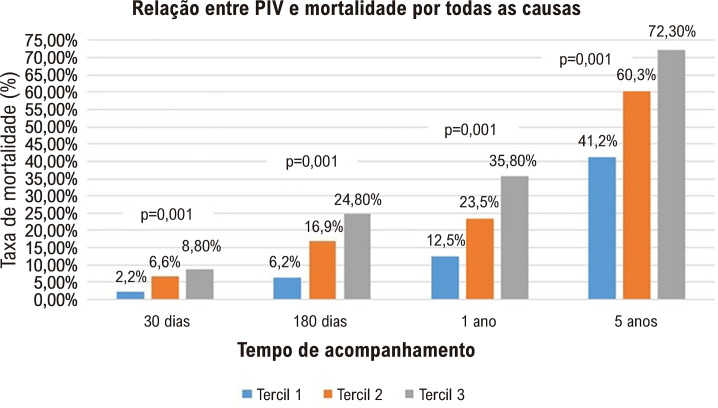
– Curva Kaplan-Meier de sobrevida global em pacientes com diferentes tercis de PIV. PIV: valor pan-imune-inflamatório.

A Figura 2 mostra que um PIV mais elevado foi associado a uma mortalidade mais elevada, tanto no curto prazo como no período de acompanhamento de 5 anos. A Figura 3 demonstra a curva de Kaplan-Meier entre os tercis dos diferentes valores de PIV. Os resultados indicaram que os pacientes com PIV mais elevado tiveram um risco aumentado de mortalidade por todas as causas a curto e longo prazo (log rank p < 0,001).

A análise de regressão logística univariada e multivariada dos pacientes é apresentada na Tabela 2. Na análise multivariada, PIV, idade, NT-proBNP e pressão sistólica da artéria pulmonar foram identificados como preditores independentes de mortalidade por todas as causas em longo prazo em pacientes com ICFEr.

**Tabela 2:** – Análises proporcionais univariadas e múltiplas de Cox para mortalidade

	Pacientes			
	Univariada		Múltipla	
	OR IC 95% da OR	Valor p	OR IC 95% da OR	Valor p
PIV Tercil 1 (Ref.)				
PIV Tercil Ref. >2	1,752 (1,247-2461)	0,001	1,565(1,055-2,323)	0,026
PIV Tercil Ref. >3	2,439(1,756-3,388)	<0,001	1,966(1,330-2,908)	0,001
Idade	1,046 (1,033–1,059)	<0,001	1,027 (1,013-1,041)	<0,001
FE	0,979 (0,963–0,995)	0,010		
K	0,860 (0,676–1,095)	0,221		
Creatinina	1,292 (1,158–1,441)	<0,001		
NT-proBNP	1,000 (1,00–1,00)	<0,001	1,001 (1,000–1,001)	<0,001
PSAP	1,018 (1,010–1,028)	<0,001	1,009 (1,00–1,019)	0,048
HGB	0,820 (0,770–0,873)	<0,001		

*IC: intervalo de confiança; FE: fração de ejeção; HGB: hemoglobina; NT-proBNP: fração N-terminal do peptídeo natriurético do tipo B; OR: razão de chances; PIV: valor pan-imune-inflamatório; PSAP: pressão sistólica da artéria pulmonar.*

## Discussão

Os principais achados do nosso estudo são os seguintes: PIV mais alto foi associado a maior mortalidade por todas as causas em curto e longo prazo em pacientes com IC agudamente descompensada, e o PIV foi um preditor independente de mortalidade por todas as causas em longo prazo em pacientes com IC agudamente descompensada. Até onde sabemos, este é o primeiro estudo a avaliar o efeito do novo biomarcador PIV no prognóstico de pacientes com IC agudamente descompensada.

Embora terapias promissoras tenham sido descobertas para o tratamento da IC nas últimas décadas, a taxa de mortalidade e morbidade ainda permanece elevada. Portanto, o desenvolvimento de novos tratamentos, bem como o desenvolvimento de biomarcadores prognósticos, é de grande importância para a estratificação de risco dos pacientes com IC. O biomarcador conhecido NT-proBNP é amplamente utilizado para avaliar o prognóstico em pacientes com IC.^15^ Embora o NT-proBNP seja um bom indicador prognóstico, ele apresenta limitações importantes na prática clínica. Primeiro, quando o NT-proBNP é comparado com o valor real, está abaixo do valor esperado devido à sua meia-vida curta. Em segundo lugar, o NT-proBNP é significativamente afetado por condições como idade, sexo e obesidade, e a sua disponibilidade não é particularmente fácil. Portanto, a busca por novos biomarcadores menos dispendiosos e mais fáceis de obter começou a atrair a atenção de pesquisadores.^16,17^

Recentemente, alguns estudos mostraram que fatores inflamatórios podem ser biomarcadores de IC^18,19^ e que alguns fatores inflamatórios estão envolvidos na ocorrência e no desenvolvimento de IC.^20,21^ No presente estudo, avaliamos a relação entre PIV e desfechos a curto e longo prazo em pacientes com IC aguda descompensada. Os resultados do nosso estudo mostraram que o PIV pode ser um biomarcador independente para mortalidade por todas as causas, a curto e longo prazo, em pacientes com IC agudamente descompensada. Em nosso estudo, verificamos que o risco de mortalidade por todas as causas aumentou ao longo do tempo em diferentes níveis de PIV. Durante o mesmo período de acompanhamento, verificamos que o risco de mortalidade por todas as causas em pacientes com IC aumentou com o aumento dos valores de PIV na admissão, incluindo mortalidade em 30 dias, 180 dias, 1 ano e 5 anos após a admissão hospitalar. De acordo com a análise de regressão de riscos proporcionais de Cox, um PIV mais elevado na admissão foi um preditor independente de mortalidade. Portanto, podemos concluir que o PIV é um poderoso biomarcador na predição do prognóstico de pacientes com IC agudamente descompensada. A análise de sobrevida de Kaplan-Meier em 5 anos mostrou que pacientes com IC agudamente descompensada e tercil de PIV mais alto tiveram sobrevida global significativamente pior.

Embora, durante anos, dados de pesquisas e estudos clínicos utilizando modelos experimentais de IC tenham apoiado o conceito de que a IC é principalmente uma doença do músculo cardíaco dominada pela ativação anormal dos sistemas neuro-hormonal e simpático, as primeiras observações clínicas que datam da década de 1950 relataram uma associação entre a proteína C reativa e diferentes etiologias de IC.^22^ Além disso, Villacorta et al. relataram que a proteína C reativa é um preditor independente de mortalidade cardiovascular em pacientes com IC aguda descompensada e que a inflamação representa um componente importante na fisiopatologia desta doença.^23^ Isso sugere que um componente inflamatório deve ser considerado na complexidade da IC. Estudos subsequentes mostraram que a inflamação desempenha um papel importante na etiologia, progressão e prognóstico da IC. Vários estudos mostraram que citocinas pró-inflamatórias elevadas, como o fator de necrose tumoral alfa (TNF-α), estavam significativamente associadas à apoptose e necrose miocárdica, provocando remodelamento ventricular adverso.^22^ Também foi demonstrado que os níveis elevados de TNF-α foram associados à função sistólica cardíaca comprometida e à baixa sobrevida em longo prazo.^24^ Além disso, descobriu-se que a interleucina (IL)-6, uma das citocinas clássicas derivadas de monócitos, estava elevada em pacientes com disfunção ventricular esquerda sem sintomas, o que sugere que é um indicador sensível para o diagnóstico precoce de IC.^25^ Markousis-Mavrogenis et al. também relataram que a IL-6 estava associada à gravidade e à sobrevida global na IC.^26^ A contagem de linfócitos, neutrófilos, monócitos e plaquetas são as principais células na resposta à infecção e à inflamação. Os neutrófilos são leucócitos que atuam como a primeira linha de defesa do hospedeiro contra patógenos e desempenham um papel importante na etiologia e no desenvolvimento da IC. Estudos anteriores demonstraram que estão associados a desfechos desfavoráveis em pacientes com IC.^27^

A RNL foi recentemente adicionada à lista de marcadores inflamatórios avaliados em pacientes com IC, após o reconhecimento do papel importante que os neutrófilos podem desempenhar no desenvolvimento e evolução da IC. Um estudo observacional mostrou que a RNL estava significativamente associada à doença renal crônica, eventos cardiovasculares maiores e rehospitalização por IC.^28^ Dois estudos recentes relataram que o índice de inflamação imune sistêmica (SII, do inglês *systemic immune-inflammation index*), que é calculado a partir da contagem de plaquetas e da RNL, foi associado a prognósticos desfavoráveis em longo prazo em pacientes com IC.^19^

O PIV é um novo biomarcador calculado a partir de neutrófilos, leucócitos, linfócitos e monócitos. Até onde sabemos, este é o primeiro estudo sobre o PIV na previsão da mortalidade na IC agudamente descompensada. O PIV foi avaliado pela primeira vez em pacientes com câncer e foi relatado como um preditor independente de mortalidade.^12,13^ Em um estudo prévio, relatamos que o PIV foi um melhor preditor de mortalidade em pacientes com infarto agudo do miocárdio com elevação do segmento ST em curto e longo prazo, em comparação com outros índices como RNL, RPL e SII.^29^

A importância do nosso estudo e a sua diferença em relação aos estudos acima mencionados é que ele inclui as 4 células mais importantes da inflamação. Os estudos prévios mostraram que os biomarcadores com mais componentes eram melhores preditores do que os biomarcadores com 1 e 2 componentes em pacientes com câncer. De Giorgi et al. mostraram que o SII tem melhor valor prognóstico que a RNL em pacientes com câncer de células renais.^30^ Por outro lado, Kucuk et al.^14^ mostraram que o PIV era melhor que o SII e a RNL no câncer colorretal metastático e no melanoma. Embora os biomarcadores anteriores baseados em hemogramas não incluíssem os monócitos, são um dos pilares mais importantes da inflamação. Além disso, estudos prévios demonstraram que eles têm um importante valor prognóstico na aterosclerose e na patogênese da IC. Também foi relatado que uma elevação nos monócitos está associada a um prognóstico desfavorável em pacientes com IC.^31^

### Limitações do estudo

Existem várias limitações no presente estudo. Primeiro, embora o período de acompanhamento de 5 anos tenha sido suficiente, o tamanho da população incluída no estudo foi pequeno. Em segundo lugar, o presente estudo foi realizado em um único centro e não validou o valor prognóstico do PIV em uma coorte de validação. Finalmente, como o estudo foi retrospectivo, apenas a mortalidade por todas as causas pôde ser avaliada, e o estudo não foi capaz de determinar o mecanismo subjacente da associação entre PIV elevado e mau prognóstico de pacientes com IC. Portanto, estudos prospectivos maiores são necessários para confirmar os resultados do presente estudo.

## Conclusão

Em conclusão, o presente estudo mostrou que valores mais elevados de PIV na admissão foram associados à mortalidade por todas as causas em 30 dias, 180 dias, 1 ano e 5 anos; portanto, o PIV pode ser usado como um preditor de prognóstico simples, de baixo custo e repetível em pacientes com IC.
