# Supplementary material for: Associação do Valor Pan-Imune-Inflamatório com Desfechos de Longo Prazo na Insuficiência Cardíaca Agudamente Descompensada
Source: Arq Bras Cardiol. 2024 Jul 1;121(6):e20230817. [Article in Portuguese] doi: 10.36660/abc.20230817 (PMC12080690; doi:10.36660/abc.20230817)
Supplement: Translation

# Translation: Association of Pan Immune-Inflammation Value with Long Term Outcomes of Acute Decompensated Heart Failure

**DOI:** 10.36660/abc.20230817i

**Authors:** Bektas Murat, Selda Murat, Mehmet Eren Altınbas, Halit Emre Yalvac, Fatih Enes Durmaz, Kadir Ugur Mert, Yüksel Cavusoglu

**Affiliations:** 1 Turkey Eskisehir City Hospital, Department of Cardiology, Eskisehir – Turkey; 2 Turkey Eskisehir Osmangazi University, Medicine Faculty Department of Cardiology, Eskisehir – Turkey; 3 Turkey Sivas Numune Hospital, Department of Cardiology, Sivas – Turkey

**Keywords:** Mortality, Biomarkers, Systolic Heart Failure

## Abstract

**Background:**

Although there have been significant improvements in the treatment of heart failure (HF) in recent decades, its prognosis remains poor. Although there are many biomarkers that can help predict the prognosis of patients with HF, there is a need for simpler, cheaper, and more easily available biomarkers.

**Objective:**

To evaluate the predictive value of pan-immune-inflammation value (PIV) in patients with acute decompensated HF.

**Methods:**

We analyzed 409 patients with HF with reduced ejection fraction who were hospitalized for acute decompensated HF. Patients were divided into 3 groups according to tertiles of PIV: tertile 1 (PIV < 357.25), tertile 2 (PIV ≥ 357.25 and < 834.55), and tertile 3 (PIV ≥ 834.55). P values < 0.05 were considered statistically significant. Kaplan-Meier curves and Cox proportional hazards regression models were used to evaluate the association between PIV and all-cause mortality. The primary outcome was 5-year all-cause mortality, and the secondary outcomes were in-hospital 30 days,, 180-day, and 1-year all-cause mortality.

**Results:**

We showed that higher PIV value was associated with both primary and secondary outcomes. The Kaplan-Meier curve showed that patients with higher PIV values had an increased risk of short- and long-term all-cause mortality (log-rank p < 0.001). In the multivariate analysis, PIV was identified as an independent predictor of long-term all-cause mortality in patients with acute decompensated HF, and we observed a 1.96-fold increase in the hazard of an event (odds ratio: 1.96, 95% confidence interval: 1.330 to 2.908, p = 0.001).

**Conclusions:**

Our study showed that the novel biomarker PIV can be used as a predictor of prognosis in patients with acute decompensated HF.

## Introduction

Heart failure (HF) is a clinical syndrome that is accompanied by symptoms such as breathlessness, ankle swelling, fatigue, and decreased activity tolerance, resulting in reduced cardiac output as a consequence of structural and/or functional impairment of the heart to maintain the perfusion and metabolic needs of various tissues and organs.^1,2^ Although improvements in treatments and their implementation have improved the survival and hospitalization of patients with heart failure with reduced ejection fraction (HFrEF) in the last 30 years, it continues to be a serious public health problem and to pose a serious economic burden, in relation to longer life expectancy and an aging global population.^3,4^

Prognosis estimation for clinical outcomes such as morbidity, mortality, and hospitalization plays an important role in helping patients, their families, and clinicians decide on the appropriate type and timing of treatment (especially decisions regarding the rapid transition to further treatments). In recent decades, although numerous prognostic markers have been identified to predict death and/or HF hospitalization for different patient populations with HF, some of them are useful in predicting death, but they fall short in predicting hospitalizations. Moreover, their clinical applicability is limited, and precise risk stratification in HF remains difficult.^5-7^

Although the specific pathogenesis of HF remains unclear, abnormal immune activation and chronic inflammation play an important role, and it has been proven that there is a close relationship between inflammation and cardiovascular diseases.^8^ Studies have shown that inflammation plays an important role in the initiation and progression of atherosclerosis and is closely associated with the pathogenesis of HF and cardiac remodeling.^9^ Growing evidence also proposes that immunological and inflammatory responses may play a pathogenic role in the development of chronic HF.^10^

Acute HF may be the first manifestation of HF (new onset) or, more often, may be due to acute decompensation of chronic HF. Acute decompensated HF is the leading cause of hospitalization in people over 65 years of age and is associated with high rates of mortality and rehospitalization, with in-hospital mortality of 4% to 10%. Compared to patients with acute decompensated HF, patients with new-onset HF may have higher in-hospital mortality, but they have lower post-discharge mortality and rehospitalization rates.^11^

More recently, a biomarker of extensive inflammation cells called pan-immune-inflammatory value (PIV), which includes neutrophil, platelet, monocyte, and lymphocyte counts has been shown to be strongly associated with worse outcomes and mortality in many cancer types. All of these studies have shown that PIV is a more stable and better indicator of inflammation than well-established immune biomarkers, such as neutrophil-to-lymphocyte ration (NLR) and platelet-to-lymphocyte ratio (PLR).^12-14^

Therefore, in this study we aimed to evaluate the long-term prognostic value of PIV, a new, low-cost, and easily available biomarker containing all immune-inflammatory components obtained from peripheral blood, in patients with acute decompensated HF.

## Methods

### Study population

This retrospective study included a total of 409 patients who were hospitalized for acute decompensated HF in our tertiary reference hospital between January 2015 and January 2020. We included patients older than 18 years old who were admitted to the emergency department and hospitalized for acute decompensated HFrEF based on European Society of Cardiology Guidelines definition.^3^ The exclusion criteria were as follows: dyspnea primarily due to non-cardiac causes, septic shock, acute coronary syndrome, pregnant women, patients with active inflammatory diseases, patients with active infection (pneumonia, urinary tract infection, etc.), patients with any leukemia, patients without hematological parameters, and HF patients hospitalized primarily due to infection (Figure 1).

**Figure 1:** – Patient inclusion flowchart.

### Data collection

Demographic data and medical history of the patients such as age, sex, hypertension, diabetes mellitus, hyperlipidemia, and smoking were collected from the hospital medical system. At the time of hospital admission, all venous blood samples routinely measured the following: glucose, creatinine, cholesterol, high-density lipoprotein cholesterol, low-density lipoprotein, total cholesterol, triglycerides, peak creatinine kinase-myocardial band, peak troponin T level, N-terminal pro-B-type natriuretic peptide (NT-proBNP), hemoglobin, neutrophil count, lymphocyte count, monocyte count, and platelet count values. All demographic, medical, echocardiographic, and laboratory data were retrieved from the hospital database. Comorbidities were accepted as diagnoses if they were found in the patient’s medical history or in the patient’s files and records.

### Calculation of pan-immune-inflammation value

PIV was calculated as: [neutrophil count (10^3^/μL) × platelet count (10^3^/μL) × monocyte count (10^3^/μL)]/ lymphocyte count (10^3^/μL).^13^

### Study outcomes

The long-term (5 years) all-cause mortality of patients with acute decompensated HF was selected as the primary outcome, while the secondary outcome was defined as short-term mortality, including the in-hospital, 30-day, 180-day, and 1-year all-cause mortalities. The primary and secondary follow-up outcome was obtained from the hospital database and telephone calls with the patients and/or their relatives.

This study received approval from our university ethics committee.

### Statistical analysis

Statistical analysis was performed using IBM SPSS Statistics 21.0 (IBM Corp., released 2012, IBM SPSS Statistics for Windows, Version 21.0. Armonk, NY, USA).

Normally distributed continuous variables are reported as mean ± standard deviation, while skewed variables are expressed as medians and interquartile ranges. Shapiro-Wilk tests were performed, and density maps were drawn to determine the normality of the distribution of the continuous variables. Categorical variables were expressed as absolute and relative frequencies, and associations related to categorical variables were verified using chi-square or Fisher’s exact test.

Comparisons of differences between groups were made by analysis of variance (one-way ANOVA). For post-hoc analysis, Tukey’s test was used. The Kruskal-Wallis H test was used to compare the groups that did not conform to the normal distribution. For post-hoc analysis, Dunn’s test was used.

Patients were grouped into 3 tertiles with increased PIV levels for further analysis.

Univariate and multivariate models were applied to predict all-cause mortality. Variables with p < 0.05 in the univariate model were included in the multivariate model to evaluate the comprehensive effects of PIV on the endpoint event. We also showed the relationship between PIV and patients’ survival through the Kaplan-Meier curve, and we used the log-rank test for hypothesis testing. The odds ratio (OR) and its 95% confidence interval (CI) were calculated. All comparisons were 2-tailed, with p < 0.05 considered significant.

## Results

According to the inclusion criteria mentioned above, 409 patients diagnosed with HF were enrolled to this study. The mean age was 62.2 ± 11.8 years, and male patients were predominant, with 279 (68.2%). The patients were divided into 3 groups (tertile 1, tertile 2, and tertile 3) according to the tertiles of PIV (Central Illustration). The baseline characteristics of the study population are listed in Table 1. Older patients had higher PIV (p = 0.038). Comparing the comorbidities among 3 groups, PIV did not show a statistical difference between hypertension and diabetes mellitus (p = 0.717 and p = 0.348, respectively). Patients with higher PIV were associated with higher rates of renal failure and chronic pulmonary disease (p = 0.002 and p = 0.004, respectively), and the prevalence of coronary artery disease was lower in the highest PIV tertile (p = 0.021).

**Table 1:** – Baseline characteristics of the study population by PIV tertile groups

	PIV < 357.25 (n=136) (1)	PIV ≥ 357.25 and < 834.55 (n=136) (2)	PIV ≥ 834.55 (n=137) (3)	p value	Multiple comparisons
Age, years	66.2±11.9	66.3±12.9	69.2±10.2	0.038	1-3: 0.041
Female, n (%)	49 (36.0%)	37 (27.2 %)	44 (32.1%)	0.293	-
HT, n (%)	83 (61.0 %)	77 (56.60%)	83 (60.6%)	0.717	-
DM, n (%)	52 (38.2%)	62 (45.6%)	63 (46.0%)	0.348	-
CAD, n (%)	82 (60.3%)	103 (75.7%)	96 (70.1%)	0.021	-
CPD, n (%)	28(20.6%)	38(27.9%)	53 (38.7%)	0.004	-
AF, n (%)	30 (22.1%)	26 (19.4%)	48 (35.0%)	0.038	-
Renal failure, n (%)	60 (44.1%)	67 (49.3%)	89.0 (65.0%)	0.002	-
HR, beats/minute	72.9±14.7	77.2±19.1	83.5±18.5	<0.001	1-3: <0.001 2-3: 0.009
Signs of hypoperfusion, n (%)	9(6.6%)	14(10.3%)	23(16.8%)	0.027	-
**Laboratory findings**					
Hemoglobin, g/dl	12.9±2.07	12.3±2.0	11.9±1.8	<0.001	1-3: <0.001
Platelet, (103 /mL)	181.0(15.25-217.75)	207.0(173.75-253.5)	250.0(194.0-303.5)	<0.001	1-2: 0.012 1-3: <0.001 2-3: <0.001
WBC, (103/mL)	7.0(5.8-8.4)	8.1(6925-9.3)	10.2(8.8-12.25)	<0.001	1-2: 0.031 1-3: <0.001 2-3: <0.001
Lymphocyte (103 /mL)	1.7(1.4-2.2)	1.45(1.0-1.8)	1.2(0.95-1.6)	<0.001	1-2: <0.001 1-3: <0.001
Neutrophil (103 /mL)	4.25(3.5-5.3)	5.7(4.625-6.0)	7.90(6.55-9.70)	<0.001	1-2: 0.007 1-3: <0.001 2-3: <0.001
Monocyte (103 /mL)	0.55(0.4250-0.70)	0.60(0.50-0.80)	0.90(0.70-1.10)	<0.001	1-3: <0.001 2-3: <0.001
LDL-C (mg/dL)	105(77.0-130.02)	95.0(73.0-121.0)	90.0(70.0-112.0)	0.010	1-3: 0.003
Triglycerides (mg/dL)	113.0(87.1-149.2)	108.0(77.65-155.25)	108(84.25-145.0)	0.778	NS
NT-proBNP, pg/ml	1828.0 (650.2-5732.0)	3949(1228.7-11308)	5108.0 (2615.2-14394.0)	<0.001	1-2: 0.016 1-3: <0.001
Albumin, g/dl	3.83±0.57	3.73±0.58	3.54±0.57	<0.001	1-3: <0.001 2-3: 0.028
CRP, (mg/dL)	5.82 (3.4-19.3)	13.65(5.64-32.5)	21.0(10.1-50.3)	<0.001	1-3: <0.001 2-3: 0.006
Creatinine, (mg/dL)	1.23±0.69	1.49±1.08	1.54±0.82	0.002	1-2: 0.044 1-3: 0.012
Glucose, (mg/dL)	107 (84-152)	112.0(88.0-153.0)	117(90.5-164.5)	0.809	NS
Sodium, (mmol/L)	139.1±3.9	138.7±4.2	136.4±5.6	<0.001	1-3: <0.001 2-3: <0.001
Potassium, (mmol/L)	4.54±0.5	4.50±0.49	4.48±0.59	0.676	NS
Troponin-T	0.022(0.013-0.05)	0.036(0.019-0.062)	0.045(0.024-0.098)	0.176	NS
LVEF, %	26.5±7.25	24.5±8.5	25.2±7.9	0.091	NS
sPAP, mm Hg	48.6±17.1	50.6±15.6	50.6±15.8	0.224	NS
**Endpoints (all-cause death)**					
30-day mortality	3 (2.2%)	9(6.6%)	12(8.8%)	<0.001	-
365-day mortality	17 (12.5%)	32(23.5%)	46(35.8%)	<0.001	-
5-year mortality	56 (41.2%)	82 (60.3%)	99 (72.3%)	<0.001	-

*AF: atrial fibrillation; CAD: coronary artery disease; CPD: chronic pulmonary disease; CRP: C-reactive protein; DM: diabetes mellitus; HR: heart rate; HT: hypertension; LDL: low-density lipoprotein; LVEF: left ventricular ejection fraction; NS: not significant; NT-proBNP: N-terminal proBNP; PIV: pan-immune-inflammation value; sPAP: systolic pulmonary artery pressure; WBC: white blood cell count.*

In the overall study population, the mean follow-up was 5 years. We observed a total of 56 events in tertile 1, 82 events in tertile 2, and 99 events in tertile 3 during the follow-up.

Figure 2 shows that a higher PIV is associated with higher mortality at both the short term and 5-year follow-up periods. Figure 3 demonstrates the Kaplan-Meier curve between the tertiles of different PIV values. The results indicated that patients with higher PIV had an increased risk of short and long-term all-cause mortality (log rank p < 0.001).

**Figure 2:** – Relationship between PIV and all-cause mortality for time periods. PIV: pan-immune-inflammation value.

**Figure 3:** – Kaplan-Meier survival curve of overall survival in patients with different PIV tertiles. PIV: pan-immune-inflammation value.

The univariate and multivariate logistic regression analysis of the patients are presented in Table 2. In the multivariate analysis, PIV, age, NT-proBNP, and systolic pulmonary artery pressure were identified as independent predictors of long-term all-cause mortality in patients with HFrEF.

**Table 2:** – Univariate and multiple Cox proportional analyses for mortality

	Patients			
	Univariate		Multiple	
	OR 95% CI of OR	p value	OR 95% CI of OR	p value
PIV Tertile 1 (Ref.)				
PIV Tertile Ref. >2	1.752 (1.247-2461)	0.001	1.565(1.055-2.323)	0.026
PIV Tertile Ref. >3	2.439(1.756-3.388)	<0.001	1.966(1.330-2.908)	0.001
Age	1.046 (1.033–1.059)	<0.001	1.027 (1.013-1.041)	<0.001
EF	0.979 (0.963–0.995)	0.010		
K	0.860 (0.676–1.095)	0.221		
Creatinine	1.292 (1.158–1.441)	<0.001		
NT-proBNP	1.000 (1.00–1.00)	<0.001	1.001 (1.000–1.001)	<0.001
sPAP	1.018 (1.010–1.028)	<0.001	1.009 (1.00–1.019)	0.048
HGB	0.820 (0.770–0.873)	<0.001		

*CI: odds ratio; EF: ejection fraction; HGB: hemoglobin; NT-proBNP: N-terminal proBNP; OR: odds ratio; PIV: pan-immune-inflammation value; sPAP: systolic pulmonary artery pressure.*

## Discussion

The main findings of our study are as follows: higher PIV was associated with higher short- and long-term all-cause mortality in patients with acute decompensated HF, and PIV was an independent predictor of long-term all-cause mortality in patients with acute decompensated HF. To our knowledge, this is the first study to evaluate the effect of the novel biomarker PIV on the prognosis of patients with acute decompensated HF.

Although promising therapies have been discovered for the treatment of HF in recent decades, the rate of mortality and morbidity still remains high. Therefore, the development of new treatments, as well as the development of prognostic biomarkers, is of great importance for risk stratification of patients with HF. The well-known biomarker NT-proBNP is widely used for evaluating prognosis in patients with HF.^15^ Although NT-proBNP is a good prognostic indicator, it has important limitations in clinical practice. First, when NT-proBNP is compared to the real value, it is below the expected value due to its short half-life. Second, NT-proBNP is significantly affected by conditions such as age, sex, and obesity, and it is not particularly easily available. Therefore, the search for new biomarkers that are cheaper and easier to obtain has begun to attract the attention of researchers.^16,17^

Recently, some studies have showed that inflammatory factors can be biomarkers of HF^18,19^ and that some inflammatory factors are involved in the occurrence and development of HF.^20, 21^ In the present study, we evaluated the relationship between PIV and short- and long-term outcomes of patients with acute decompensated HF. The results of our study showed that PIV could an independent biomarker for short- and long-term all-cause mortality of patients with acute decompensated HF. In our study we found that the risk of all-cause mortality increased over time at different PIV levels. During the same follow-up period, we found that the risk of all-cause mortality in patients with HF increased with increasing PIV values at admission, including mortality at 30 days, 180 days, 1 year, and 5 years after admission to the hospital. According to Cox proportional hazards regression analysis, a higher PIV at admission was an independent predictor of mortality. Therefore, we can conclude that PIV is a powerful biomarker in predicting the prognosis of patients with acute decompensated HF. Five-year Kaplan-Meier survival analysis showed that acute decompensated HF patients with higher PIV tertile had significantly poorer overall survival.

Although, for years, data from research and clinical studies using experimental HF models have supported the concept that HF is primarily a heart muscle disease dominated by abnormal activation of the neurohormonal and sympathetic systems, early clinical observations dating back to the 1950s reported an association between C-reactive protein and different etiologies of HF.^22^ Additionally, Villacorta et al. reported that C-reactive protein is an independent predictor of cardiovascular mortality in patients with acute decompensated HF and that inflammation represents an important component in the pathophysiology of this disease.^23^ This suggested that an inflammatory component should be considered in the complexity of HF. Later studies showed that inflammation has an important role in the etiology, progression, and prognosis of HF. Several studies showed that elevated pro-inflammatory cytokines such as tumor necrosis factor-alpha (TNF-α) were significantly associated with myocardial apoptosis and necrosis, bringing about ventricular adverse remodeling.^22^ In addition, it was shown that the elevated levels of TNF-α were associated with impaired cardiac systolic function and poor long-term survival.^24^ Also, interleukin (IL)-6, one of the classical cytokines derived from monocytes, was found to be elevated in patients with left ventricular dysfunction without symptoms, and it has been suggested that it is a sensitive indicator for early diagnosis of HF.^25^ Markousis-Mavrogenis et al. also reported that IL-6 was associated with severity and overall survival in HF.^26^ Lymphocyte, neutrophil, monocytes, and platelet counts are the main cells in the response to infection and inflammation. Neutrophils are leukocytes that act as the first line of host defense against pathogens and play an important role in the etiology and development of HF. Previous studies have shown that they is associated with poor outcome in HF patients.^27^

The NLR has recently been added to the list of inflammatory markers evaluated in HF patients, following the emergence of the important role that neutrophils can play in the development and outcome of HF. An observational study showed that NLR was significantly associated with chronic kidney disease, major cardiovascular events, and re-hospitalization for HF.^28^ Two recent studies reported that systemic immune-inflammation index (SII), which is calculated from platelet counts and NLR, was associated with poor long-term prognoses in patients with HF.^19^

PIV is a novel biomarker that is calculated from neutrophils, leukocytes, lymphocytes, and monocytes. To the best of our knowledge, this is the first study on PIV in predicting mortality of acute decompensated HF. PIV was first evaluated in cancer patients, and it has been reported to be an independent predictor of mortality.^12,13^ In a previous study, we reported that PIV was a better predictor of mortality in patients with acute ST-segment elevation myocardial infarction in the short and long term, compared to other indexes such as NLR, PLR, and SII.^29^

The importance of our study and its difference from the abovementioned studies are that it contains the 4 most important cells of inflammation. The previous studies showed that biomarkers with more components were better predictors than biomarkers with 1 and 2 components in cancer patients. De Giorgi et al. showed that SII has a better prognostic value than NLR in patients with renal cell cancer.^30^ On the other hand, Fuca et al. showed that PIV was better than SII and NLR in metastatic colorectal cancer and melanoma.^14^Although the previous blood count-based biomarkers did not include monocytes, they are one of the most important cornerstones of inflammation. In addition, previous studies have shown that they have an important prognostic value in atherosclerosis and HF pathogenesis. It has also been reported that an elevation in monocytes is associated with poor prognosis in patients with HF.^31^

### Study limitations

There are several limitations in this study. First, although the follow-up period was sufficient with 5 years, the size of the population included in the study was small. Second, this study was conducted at a single center and did not validate the prognostic value of PIV in a validation cohort. Finally, because the study was retrospective, only all-cause mortality could be evaluated, and the study was unable to determine the underlying mechanism of the association between high PIV and poor prognosis of patients with HF. Therefore, larger prospective studies are needed to confirm the results of this study.

## Conclusion

In conclusion, this study showed that higher PIV values at admission were associated with 30-day, 180-day, 1-year, and 5-year all-cause mortality; therefore, PIV can be used as a low-cost, simple, and repeatable predictor of prognosis in patients with HF.
